# Association between plasminogen activator inhibitor-1 and cardiovascular events: a systematic review and meta-analysis

**DOI:** 10.1186/s12959-018-0166-4

**Published:** 2018-06-05

**Authors:** Richard G. Jung, Pouya Motazedian, F. Daniel Ramirez, Trevor Simard, Pietro Di Santo, Sarah Visintini, Mohammad Ali Faraz, Alisha Labinaz, Young Jung, Benjamin Hibbert

**Affiliations:** 1 CAPITAL Research Group, University of Ottawa Heart Institute, 40 Ruskin Street, H-4238, Ottawa, ON K1Y 4W7 Canada; 2 Department of Cellular and Molecular Medicine, University of Ottawa, Ottawa, ON Canada; 3 Vascular Biology and Experimental Medicine Laboratory, University of Ottawa Heart Institute, Ottawa, ON Canada; 4 Division of Cardiology, University of Ottawa Heart Institute, Ottawa, ON Canada; 5 School of Epidemiology, Public Health and Preventive Medicine, University of Ottawa, Ottawa, ON Canada; 6 Berkman Library, University of Ottawa Heart Institute, Ottawa, ON Canada; 7 Department of Health Research Methods, Evidence, and Impact, McMaster University, Hamilton, ON Canada

**Keywords:** Plasminogen activator inhibitor-1, Biomarkers, Mortality, Myocardial infarction, Meta-analysis

## Abstract

**Background:**

Small studies have implicated plasminogen activator inhibitor-1 (PAI-1) as a predictor of cardiovascular events; however, these findings have been inconsistent.

We sought out to examine the potential role of PAI-1 as a marker for major adverse cardiovascular events (MACE).

**Methods:**

We systematically reviewed all indexed studies examining the association between PAI-1 and MACE (defined as death, myocardial infarction, or cerebrovascular accident) or restenosis. EMBASE, Web of Science, Medline, and the Cochrane Library were searched through October 2016 to identify relevant studies, supplemented by letters to authors and review of citations. Studies reporting the results of PAI-1 antigen and/or activity levels in association with MACE in human subjects were included.

**Results:**

Of 5961 articles screened, we identified 38 articles published between 1991 to 2016 that reported PAI-1 levels in 11,557 patients. In studies that examined PAI-1 antigen and activity levels, 15.1% and 29.6% of patients experienced MACE, respectively. Patients with MACE had higher PAI-1 antigen levels with a mean difference of 6.11 ng/mL (95% CI, 3.27-8.96). This finding was similar among patients with and without known coronary artery disease. Comparatively, studies that stratified by PAI-1 activity levels were not associated with MACE. In contrast, studies of coronary restenosis suggest PAI-1 antigen and activity levels are negatively associated with MACE.

**Conclusions:**

Elevated plasma PAI-1 antigen levels are associated with MACE. Definitive studies are needed to ascertain if PAI-1 acts simply as a marker of risk or if it is indeed a bona fide therapeutic target.

**Electronic supplementary material:**

The online version of this article (10.1186/s12959-018-0166-4) contains supplementary material, which is available to authorized users.

## Background

Obstructive coronary artery disease (CAD) is the leading cause of mortality in the western world. The cornerstone of therapy for CAD remains revascularization and secondary medical therapy to modify risk factors. The fibrinolytic system has implications for both approaches to disease management. First, percutaneous coronary intervention (PCI) with implantation of a coronary stent remains the predominant method of coronary revascularization [1]. However, complications such as in-stent restenosis and stent thrombosis following PCI limit its efficacy. Thus, in the peri-revascularization period, preventing thrombotic events is paramount until the vessel’s endothelial lining and function are restored. Second, long term therapy with antiplatelet and/or oral anticoagulation is an integral part of secondary preventive medical therapy. Thus, dysregulation of the fibrinolytic pathways may increase the risk of complications from revascularization therapy *and* diminish the efficacy of long term medical therapy to reduce the risk of recurrent events.

The fibrinolytic system is activated by the conversion of plasminogen to plasmin by serine proteases such as tissue or urokinase-type plasminogen activator (t-PA and u-PA, respectively). In contrast, fibrinolysis is inhibited by plasminogen activator inhibitor-1 (PAI-1), which is a member of the serine protease inhibitor (serpin) family. Ultimately, thrombosis risk is influenced by the balance between PAI-1 and t-PA. Thus, an increase in the PAI-1 levels in plasma can induce a hypercoagulable state [2]. PAI-1 is released by vascular endothelial cells, hepatocytes, adipocytes, cardiomyocytes, fibroblasts, and platelets [3, 4]. In healthy humans, plasma levels of PAI-1 exceed t-PA by a ratio of over 4:1 with most of PAI-1 being cleared by the liver [5]. In pathologic conditions, PAI-1 production can be upregulated by pro-inflammatory factors such as TNF*α*, TGF*β*, and insulin [6]. Elevated plasma PAI-1 levels have been associated with impaired fibrinolytic activity in stroke and coronary artery disease [7]. Moreover, PAI-1 antigen and activity levels are elevated in patients with type 2 diabetes [8], hyperinsulinemia [9], and those with insulin resistance [10, 11]. Yet, a definitive assessment of the impact of elevated PAI-1 as a biomarker or therapeutic target has yet to be evaluated. Accordingly, we performed a systematic review and meta-analysis of PAI-1 antigen and activity levels and their relationship with major adverse cardiovascular events (MACE) in humans.

## Methods

### Literature search strategy

Literature searches were guided by a medical librarian with expertise in systematic reviews (S.V.) using a combination of key terms and index headings related to PAI-1, coronary disease (informed by the Cochrane review search strategy for coronary heart disease in exercise-based cardiac rehabilitation [12]), and the Cochrane Highly Sensitive search strategy to eliminate articles on animal studies in Medline. The search was additionally peer-reviewed by a second medical librarian (R.S.). Once finalized, the search strategy was then translated to other bibliographic databases (see Additional file 1 for the full Medline search). The final search was conducted on October 2016 in Medline (Ovid) (In-Process & Other Non-Indexed Citations and Ovid MEDLINE(R) 1946-), Embase (Ovid) (Embase Classic + Embase 1947-), Cochrane Library (Ovid) (from inception), and Web of Science (Thomson Reuters) (all indexes, from inception). Search results were exported to EndNote X7 (Thomson Reuters, New York, USA) and duplicates eliminated using the program’s duplicate identification feature and manual inspection. A review protocol was produced but not registered in a database.

Titles and abstracts were screened by two independent reviewers (R.J. and P.M.) using Covidence (Melbourne, Australia). Full articles were retrieved in cases of missing abstracts. Corresponding authors were contacted for additional information when necessary.

### Inclusion and exclusion criteria and quality assessment

Studies were included if they met the following criteria: (1) PAI-1 antigen or activity levels were reported; (2) the population studied comprised individuals aged 18 years or older; (3) components of MACE (death, myocardial infarction, and cerebrovascular events including stroke and transient ischemic attacks) or restenosis were reported; (4) articles were published in English. Exclusion criteria included: (1) PAI-1 polymorphism studies examining the association between 4G/5G and adverse events; (2) animal or in vitro studies; and (3) studies reporting hazard ratios only. Full text data extraction was conducted by two independent evaluators (R.J. and P.M.). Each reviewer independently extracted patient population characteristics, group sizes, PAI-1 antigen and activity levels, follow-up duration, and MACE and restenosis. All discrepancies were resolved by consensus prior to locking the database for analysis (Tables 1 and 2). Included observational studies were evaluated for quality and risk of bias using the Newcastle-Ottawa Quality Assessment Scale [13] by two independent evaluators (R.J. and P.M.) with disagreements resolved by consensus. Visual funnel plot inspection was used to screen for publication bias.

**Table 1:** Studies reporting PAI-1 antigen levels (ng/mL) and major adverse cardiovascular events and restenosis

					Event								No Event	
Reference	Year	Study Design	Follow-up (months)	Population of interest	N	PAI-1 (IU/mL)	Range	Death	MI	Restenosis	CVA	N	PAI-1 (IU/mL)	Range
Sane et al. [50]	1991	Cohort	24	Fibrinolytics	24	57	58	24	n/a	n/a	n/a	315	54	53
Cortellaro et al. [51]	1993	Case-Control	24	Subgroups Combined	58	11.3	0.7	13	17	n/a	20	87	7.3	0 5
Brannstrom et al. [52]	1995	Cohort	46	Anticoagulant	38	21.9	15.1	38	n/a	n/a	n/a	167	16.7	11.8
Juhan-Vague et al. [53]	1996	Cohort	24	MI	106	18.2	8 2	40	66	n/a	n/a	2700	14.8	8.9
Nordt et al. [54]	1998	Cohort	12	Fibrinolytics	5	28.8	14.3	n/a	n/a	5	n/a	26	27.1	15.4
Alaigh et al. [55]	1998	Cohort	6	Elective PCI	28	20.75	11.06	n/a	n/a	28	n/a	45	24.5	13.85
Moss et al. [56]	1999	Cohort	26	MI	81	25	18	25	56	n/a	n/a	964	29	28
Redondo et al. [57]	2001	Cohort	24	MI	37	40.725	22.28	2	5	30	n/a	157	42.65	21.02
Fornitz et al. [58]	2001	Cohort	6	Elective PCI	7	82.6	26.6	n/a	n/a	7	n/a	12	72.2	27
Bogaty et al. [59]	2001	Case-Control	48	MI	23	23.83	19.04	n/a	8	n/a	n/a	77	18.9	16.24
Ganti et al. [60]	2002	Cohort	n/a	MI	4	80.68	16.38	4	n/a	n/a	n/a	38	61	21.95
Lip et al. [61]	2002	Cohort	12	Stroke	27	56.5	30.7	27	n/a	n/a	n/a	59	45.9	23.3
Inoue et al. [62]	2003	Cohort	6	MI	24	28	4	n/a	n/a	24	n/a	42	29	4
Christ et al. [20]	2005	Cohort	6	Elective PCI	25	14.8	0.7	n/a	n/a	25	n/a	55	16.8	2.1
Robinson et al. [63]	2007	Cohort	5 to 51	Coronary Heart Disease	19	36.3	17.9	2	2	n/a	2	79	45.4	25.6
Katsaros et al. [24]	2008	Cohort	6 to 8	Elective PCI	12	11.69	8.05	n/a	n/a	12	n/a	61	22.78	18.76
Thogersen et al. [64]	2009	Case-Control	n/a	Healthy	50	38.27	16.79	n/a	50	n/a	n/a	56	29	15.75
Akkus et al. [65]	2009	Cohort	12	Cardiogenic Shock	33	116.5	97.26	33	n/a	n/a	n/a	27	71.33	54.79
Pineda et al. [66]	2010	Cohort	36	MI	25	65.13	53.3	4	21	n/a	n/a	117	70.1	48.34
Wennberg et al. [67]	2012	Case-Control	168	Healthy	469	57.28	25.83	n/a	469	n/a	n/a	895	51.23	25.11
Yano et al. [68]	2013	Cohort	20	Smokers	66	65.73	64.57	n/a	11	n/a	55	744	42.97	36.54
Yano et al. [69]	2014	Cohort	30	Hypertension	42	31.67	16.12	4	13	n/a	16	548	28.33	14.87
Knudsen et al. [70]	2014	Case-Control	12	HIV	54	111	8.5	3	51	n/a	n/a	54	92	7
Golukhova et al. [71]	2015	Cohort	28	Elective PCI	23	72.75	29.86	2	9	11	n/a	71	49.75	23.16

*CVA* cerebrovascular accident

**Table 2:** Studies reporting PAI-1 activity levels (IU/mL) and major adverse cardiovascular events and restenosis

					Event						No Event		
Reference	Year	Study Design	Follow-up (months)	Population of interest	N	PAI-1 Activity (U/mL)	Range	Death	MI	Restenosis	N	PAI-1 Activity (U/mL)	Range
Sane et al. [50]	1991	Cohort	24	Fibrinolytics	28	17	16	n/a	n/a	28	328	19	21
Shah et al. [72]	1992	Cohort	9	Elective PCI	28	8	7.1	n/a	n/a	28	40	12	8
Gray et al. [73]	1993	Cohort	0.1	MI	13	20.6	11	n/a	13	n/a	85	20.1	7.9
Malmberg et al. [74]	1994	Case-Control	90	MI	53	23.75	13.34	20	33	n/a	55	18	10.97
Brack et al. [75]	1994	Cohort	4	Elective PCI	16	4.63	4.71	n/a	n/a	16	30	5.77	5.06
Nordt et al. [54]	1998	Cohort	12	Fibrinolytics	5	8.7	8.3	n/a	5	n/a	26	9.9	8.2
Jansson et al. [76]	1998	Cohort	120	MI	54	9.1	5.1	54	n/a	n/a	69	10.6	7.1
Wiman et al. [77]	2000	Case-Control	3	MI	61	22.1	17.5	n/a	61	n/a	95	18.2	16.5
Wiman et al. [77]	2000	Case-Control	3	Ml	25	15.4	13.6	n/a	25	n/a	38	17.8	12.4
Prisco et al. [78]	2001	Cohort	18	MI	18	11.27	7.64	n/a	n/a	18	36	15.8	27.49
Prisco et al. [78]	2001	Cohort	18	Elective PCI	6	8.33	8.1	n/a	n/a	6	42	7.57	9.52
Sargento et al. [79]	2003	Cohort	12	MI	7	6.34	1.56	5	2	n/a	80	4.47	1.84
Marcucci et al. [19]	2006	Case-Control	22 2	MI	109	22	9.09	54	55	n/a	411	24.25	10.63
Schoebel et al. [80]	2008	Cohort	2	MI	18	3.7	1.8	n/a	n/a	18	42	5.3	3.2

Wiman et al. [77] is presented twice as data for men and women were reported separately

### Statistical analysis

The primary clinical endpoint for this study was MACE – a composite of death, myocardial infarction, or cerebrovascular events. The secondary endpoint included components of the primary as well as coronary restenosis in patients undergoing coronary revascularization. Mean circulating PAI-1 antigen (ng/mL) and activity (IU/mL) levels and their associated standard deviations were used for analyses. Fourteen studies reported median and interquartile ranges, which were converted to approximated means and standard deviations using the method described by Wan et al. [14].

All analyses were performed using Review Manager (RevMan) 5.3 (Cochrane Collection, Copenhagen, Denmark). PAI-1 antigen and activity levels were compared between patients with or without the outcomes of interest either as absolute values or dichotomized as high vs. low. Random effects models stratified by study design and study quality were used to generate pooled mean differences with 95% confidence intervals. *Post-hoc* meta regression was performed to account for timing of blood draw and acute phase reactions in studies of patients presenting with acute myocardial infarction or stroke.

## Results

### Included studies

#### Study selection

**Fig. 1:**
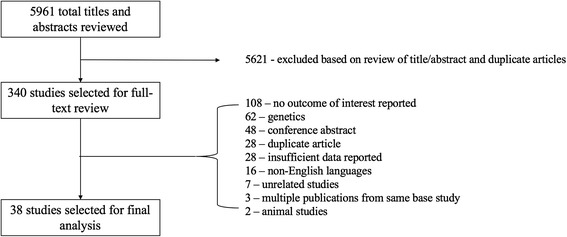
Flow diagram of the included PAI-1 studies for meta-analysis

After excluding duplicate articles, 5961 titles and abstracts were screened, of which 340 underwent full review and 38 were ultimately included (Fig. 1, Tables 1 and 2). Study populations were heterogeneous, including patients presenting with stable angina, acute coronary syndrome, and non-cardiac diseases. Study sample sizes ranged from 19 to 2806. Most studies were of moderate quality (see Additional file 1: Tables S1 and S2 for details of study quality assessments). Funnel plots are shown in Additional file 1: Figures S1 and S2. In pooled analyses of all studies reporting PAI-1 antigen levels (*n* = 8999), 1362 events were reported, including 234 deaths, 795 myocardial infarctions, 101 cerebrovascular events, and 142 restenoses (Table 1). In all studies that examined PAI-1 activity levels (*n* = 1490), 441 events were reported, including 133 deaths, 194 myocardial infarctions, and 114 cases of restenosis (Table 2).

### PAI-1 and clinical outcomes

#### Major adverse cardiovascular events

**Fig. 2:**
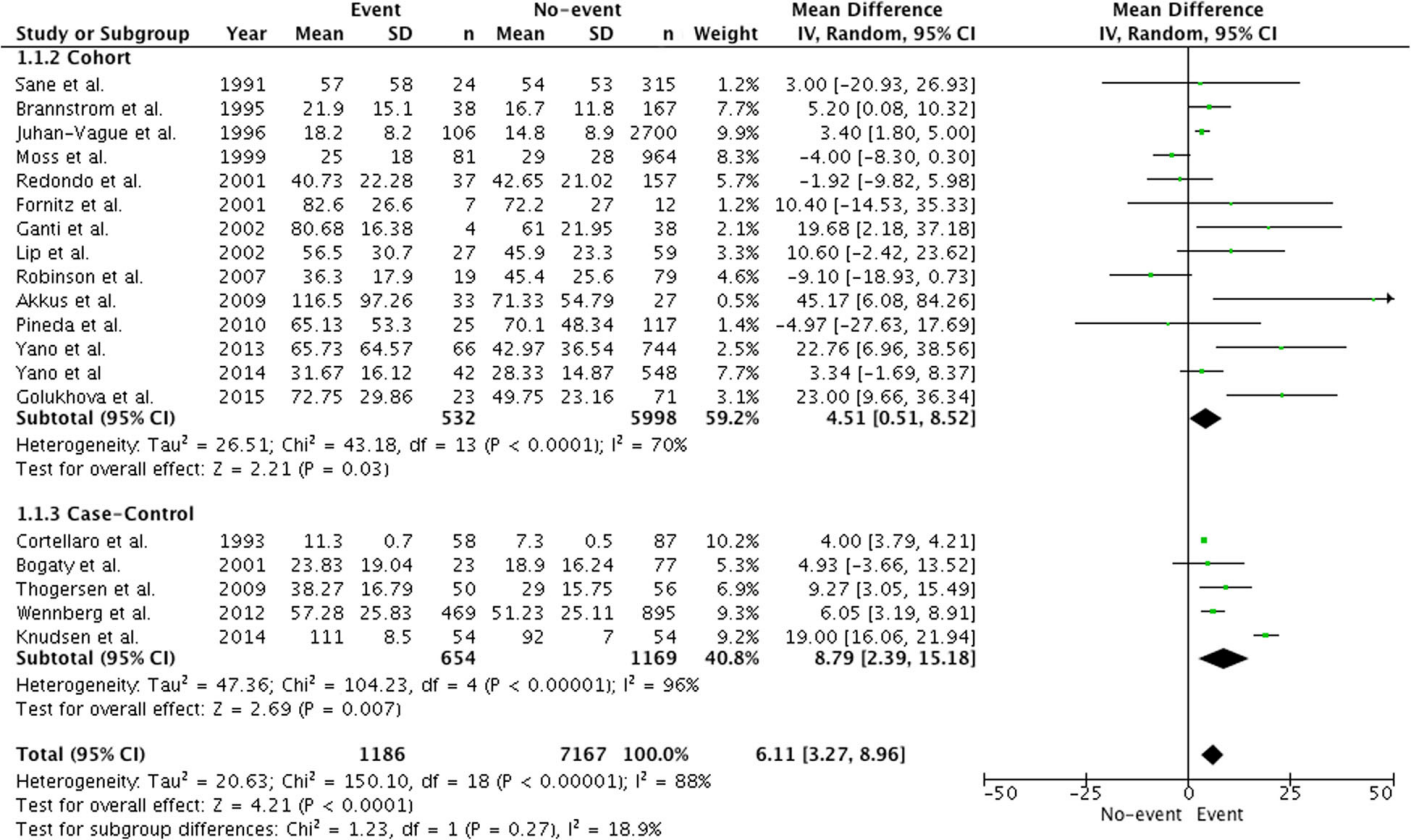
Comparison of mean PAI-1 antigen levels (ng/mL) in patients with major adverse cardiac events and control patients. Data is expressed as a mean difference and analyzed using a random effects model

PAI-1 antigen levels were higher in those with MACE with a mean difference of 6.11 ng/mL (95% CI, 3.27-8.96, *P* < 0.001) – a difference that was present irrespective of study design (Fig. 2). When restricted to high-quality studies, PAI-1 antigen levels in patients with MACE were 5.22 ng/mL (95% CI, 2.97-7.54, P < 0.001; Additional file 1: Figure S3). Among seven studies reporting morning blood draws between 7:00 and 10:00 am, PAI-1 antigen levels were higher in those with MACE with a mean difference of 4.61 ng/mL (95% CI, 1.49-7.74, *P* = 0.004; Additional file 1: Figure S4). Meta-regression analysis of timing of the blood draw and acute phase studies was not predictive of the heterogeneity in our selected studies nor did it contribute to a greater understanding of the impact of PAI-1 in its association with MACE (Additional file 1: Table S3).

**Fig. 3:**
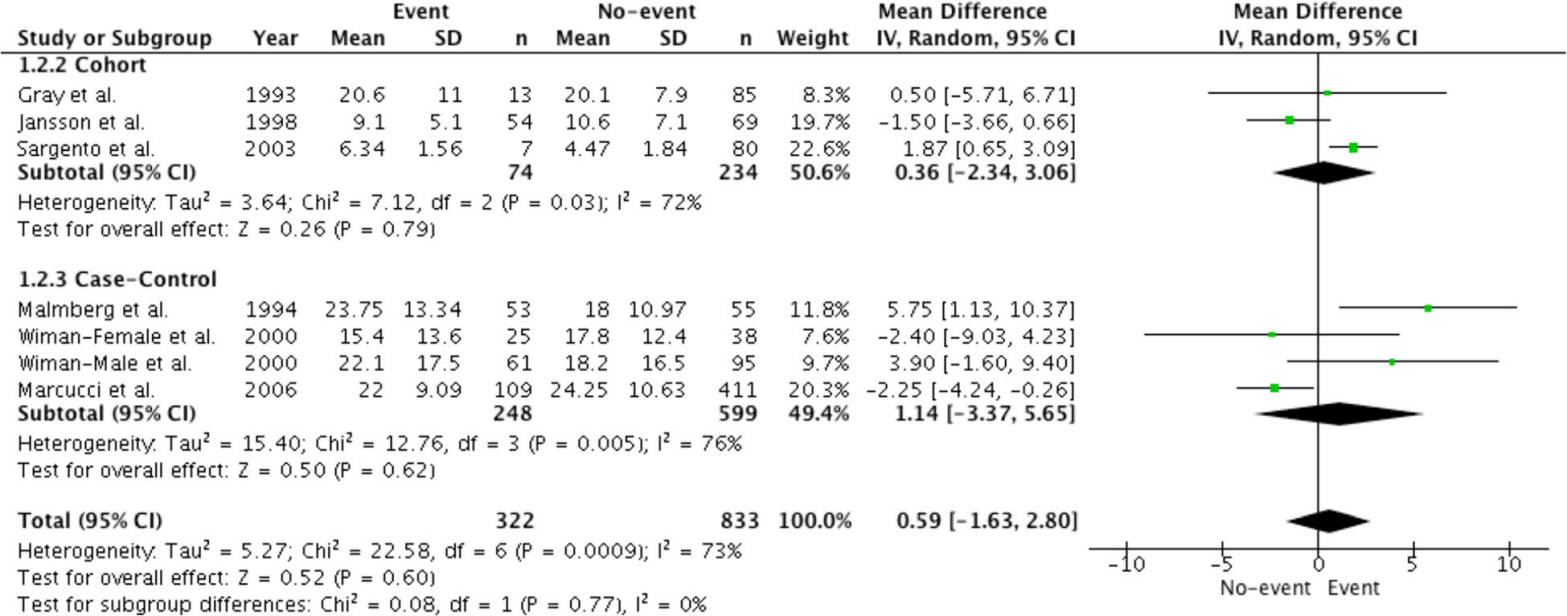
Comparison of mean PAI-1 activity levels (IU/mL) in patients with major adverse cardiac events and control patients. Data is expressed as a mean difference and analyzed using a random effects model

In contrast to PAI-1 plasma antigen levels, there was no significant difference in PAI-1 activity levels between those with vs. without MACE (mean difference 0.59 IU/mL (95% CI, − 1.63-2.80, *P* = 0.60; Fig. 3). No association between PAI-1 activity levels and MACE was observed when the analysis was restricted to three high-quality studies with a mean difference of 1.14 IU/mL (95% CI, − 3.37-5.65, *P* = 0.62; Additional file 1: Figure S5).

### Major adverse cardiovascular events: Pre-existing versus no known coronary artery disease

**Fig. 4:**
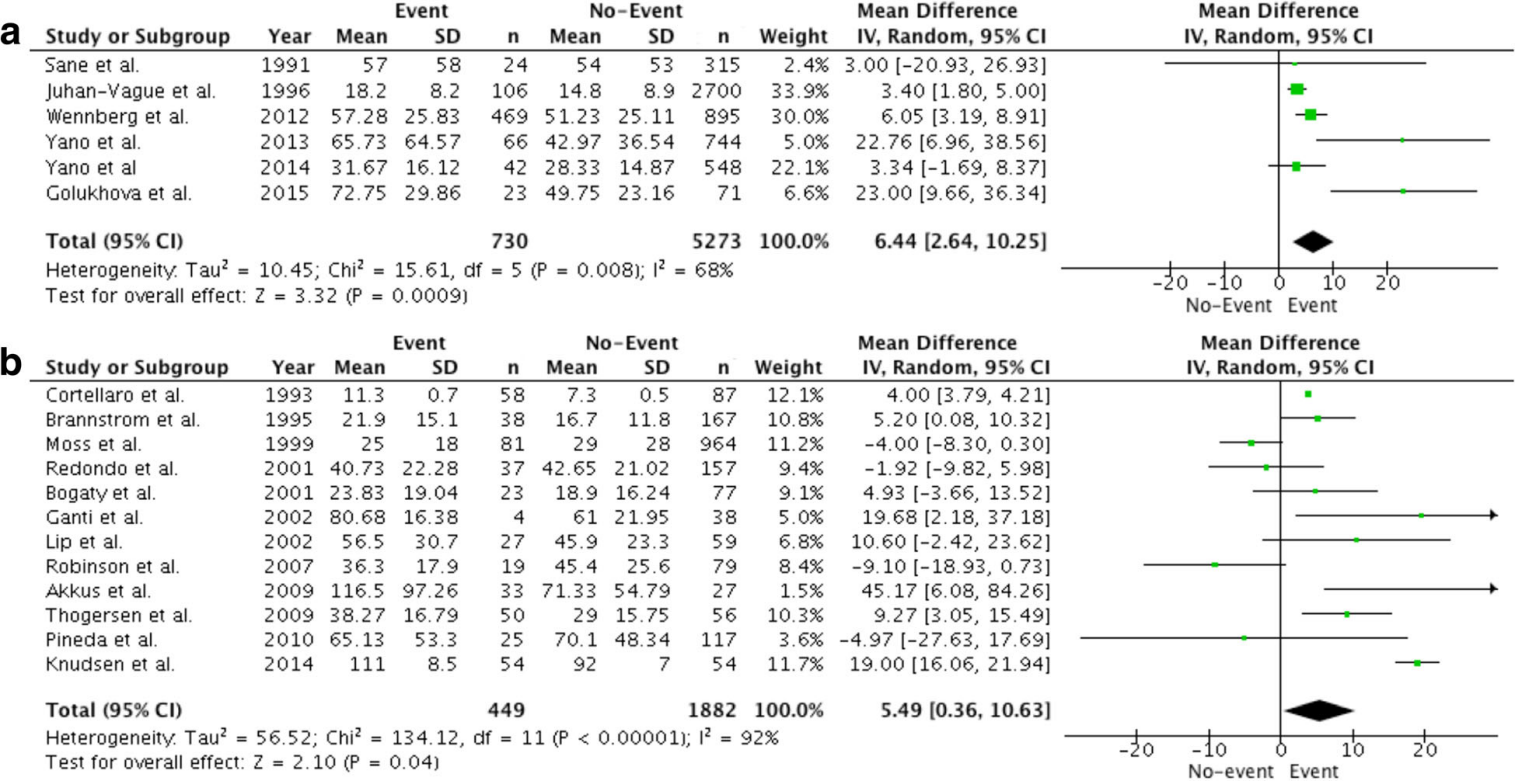
Comparison of mean PAI-1 antigen levels (ng/mL) in patients with primary and secondary major adverse cardiac events and control patients. Data is expressed as a mean difference and analyzed using a random effects model. **a** PAI-1 levels (ng/mL) in patients with primary major adverse cardiac events. **b** PAI-1 levels (ng/mL) in patients with secondary major adverse cardiac events

Overall, among patients without prior coronary artery disease, 12.2% had an event. PAI-1 antigen levels in those without previously known coronary artery disease were on average 6.44 ng/mL higher in those with MACE relative to those without (95% CI, 2.64-10.25, *P* < 0.001; Fig. 4a). In studies that included patients with known CAD, 19.3% had MACE. PAI-1 antigen levels in those with known CAD were on average 5.49 ng/mL higher in those with MACE than those without (95% CI, 0.36-10.63, *P* = 0.04; Fig. 4b). No difference in PAI-1 activity levels between event and control groups was observed in the five studies reporting PAI-1 activity levels and MACE (Additional file 1: Figure S6).

### Death

In the five studies that reported mortality data, 126 deaths were observed (17.2% of patients). PAI-1 antigen levels were higher among patients who died (mean difference: 10.34 ng/mL (95% CI, 1.90-18.79, *P* = 0.02; Additional file 1: Figure S7).

### Restenosis

**Fig. 5:**
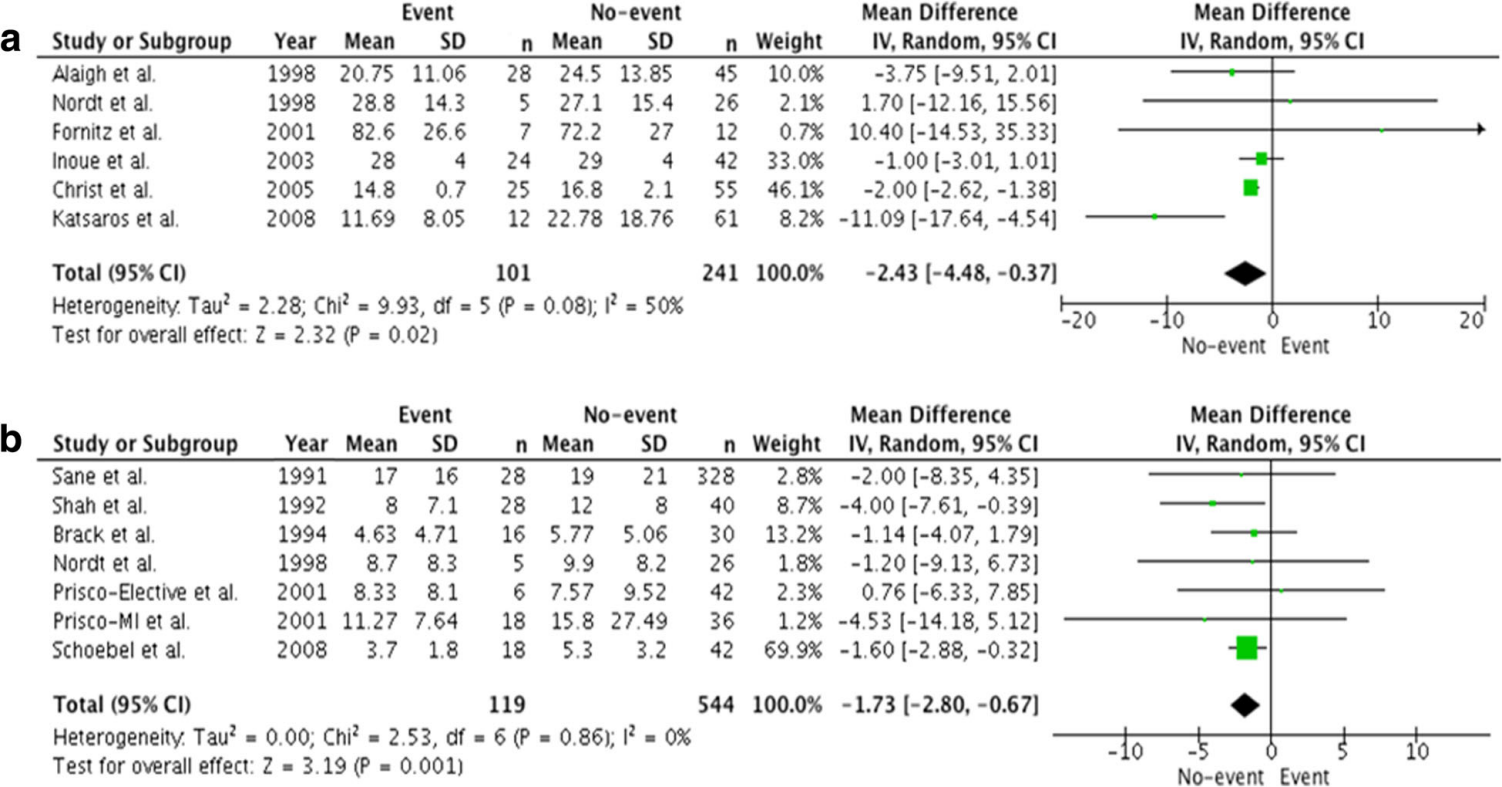
Comparison of mean PAI-1 antigen and activity levels in patients with restenosis and control patients. Data is expressed as a mean difference and analyzed using a random effects model. **a** Comparison of mean PAI-1 antigen levels (ng/mL) in patients with restenosis. **b** Comparison of mean PAI-1 activity levels (IU/mL) in patients with restenosis

In the six studies that examined restenosis following percutaneous coronary intervention (with and without coronary stent implantation), 101 events were observed (29.5% of patients). PAI-1 antigen levels were lower in those with restenosis with a mean difference of − 2.43 ng/mL (95% CI, − 4.48-(− 0.37), *P* = 0.02; Fig. 5a). An additional six studies provided restenosis rates and PAI-1 activity levels. These studies reported restenosis in 119 patients (17.9%). PAI-1 activity was lower in those with restenosis with a mean difference of − 1.73 IU/mL (95% CI: -2.80-(− 0.67), *P* = 0.001; Fig. 5b).

### High versus low PAI-1 levels

**Fig. 6:**
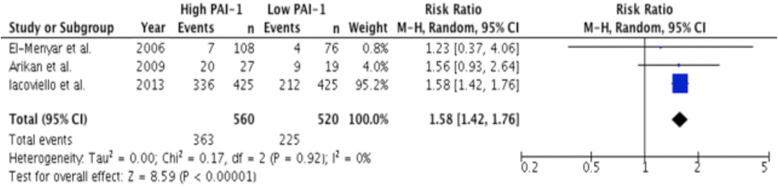
Comparison of risk of major adverse cardiac events in patients stratified by PAI-1 antigen levels (ng/mL). Data is expressed as a risk ratio and analyzed using a random effects model

Three studies stratified their data by high versus low PAI-1 antigen levels. These studies reported a MACE rate of 54.4% [15–17]. High PAI-1 antigen levels were associated with a 58% greater risk of MACE compared to low PAI-1 antigen levels (RR 1.58, 95% CI: 1.42-1.76, *P* < 0.0001; Fig. 6).

## Discussion

Incident and recurrent cardiovascular events remain important adverse outcomes despite major advances in revascularization and medical therapy. PAI-1 has been associated with MACE, but whether it is solely a marker for these events or a mediator with the potential of representing a unique therapeutic target is uncertain. Our analysis set out to evaluate the current state of evidence linking PAI-1 antigen and activity levels with these outcomes. Our study suggests that elevated PAI-1 antigen levels are associated with major adverse cardiac events in both primary and secondary event populations. In addition, elevated PAI-1 antigen levels were associated with all-cause mortality. While the populations studied were heterogeneous, the robustness of the association suggests that PAI-1 warrants further study as a marker and potential mediator of adverse cardiovascular events.

Our findings build upon the growing evidence that PAI-1 is a biomarker for MACE in patients with CAD. Tofler et al. identified elevated PAI-1 antigen levels to be predictive of cardiovascular disease [18]. In addition, previous studies of elevated PAI-1 antigen and activity levels predicted acute coronary syndrome after coronary stenting [15, 19–22]. Furthermore, Song et al. recently identified a causal relationship between elevated PAI-1 levels and incident CAD [23]. Our study expands on these findings by identifying elevated PAI-1 antigen levels as being associated with MACE in both primary (incident) and secondary event populations thereby suggesting a broader relevance of PAI-1 antigen levels. In addition, we demonstrate the potential applicability of PAI-1 antigen levels in predicting restenosis, consistent with a previous report by Katsaros et al. [24], which identified that patients with the lowest PAI-1 antigen tertile had a 9.5-fold increased risk of in-stent restenosis in patients managed with modern drug-eluting stents. However, our study, as with those mentioned above, are unable to ascertain if PAI-1 is a mediator or simply a marker of these events. Further study is needed to establish this important distinction.

The association of PAI-1 activity with MACE did not meet our pre-specified thresholds for significance. Although this finding suggests that PAI-1 antigen levels may be more robust as a biomarker, PAI-1 activity is a functional measure of the entire PAI-1 content in the plasma. The measurement of PAI-1 antigen captures the entire PAI-1 content in the sample in the form of free and active PAI-1 (which we refer to as PAI-1 activity), PAI-1 complexed to t-PA or u-PA, and latent PAI-1. Although PAI-1 antigen and activity levels are correlated, antigen levels will not necessarily reflect PAI-1 activity levels [25]. Indeed, at time of acute trauma such as plaque rupture, t-PA will complex with PAI-1 at a 1:1 ratio reducing detectable PAI-1 activity but not PAI-1 antigen levels. In addition, PAI-1 activity is influenced by experimental techniques during sample isolation such as freeze-thaw or sonication [26], low temperature, low pH, and high salt concentrations [27]. Factors which influence PAI-1 activity levels which impacted the significance of our findings include the method of PAI-1 extraction and isolation [26–30], time of blood draw [31], intra- and inter-assay variability in PAI-1 activity and antigen levels [32], and baseline risk factors which influences PAI-1 levels such as smoking [33, 34], high-fat diet [35], and maximal exercise [36]. Finally, in addition to important biological differences, manifest differences in the quality and power of studies examining antigen and activity levels existed which may explain the divergent results.

In-stent restenosis (ISR) is a result of neointimal formation or intimal thickening that narrows the vascular lumen following PCI [37]. The detailed molecular mechanism behind the pathophysiology of ISR has been reviewed elsewhere [38]. Briefly, studies have revealed that the initial recruitment of inflammatory cells is subsequently followed by smooth muscle cells (SMC) and myofibroblasts recruitment, which creates the extracellular matrix that narrows the vascular lumen [38]. SMCs achieve their peak proliferation at 48-96 h post-injury in the media and intima and return to their baseline following re-endothelialization of that artery within 8 weeks [39]. Conflicting evidence exists in the literature in the role of PAI-1 in cell migration. PAI-1 binding to low-density lipoprotein receptor-related protein 1 (LRP1) in SMCs promotes cell migration [40]. However, PAI-1 complexed to vitronectin has been demonstrated to inhibit cell migration and adhesion [41]. Thus, biological plausibility exists to link PAI-1 and restenosis following coronary intervention.

Clinically, low PAI-1 antigen and activity levels have been found to be associated with increased restenosis in our study; however, several limitations exist in these studies. First, these selected studies range from 1991 to 2008, during which time the intervention of choice evolved from balloon angioplasty to bare-metal stents to drug-eluting stents, which reduced the rate of ISR observed today [1]. Second, anti-proliferative agents that coat drug-eluting stents such as paclitaxel promote PAI-1 transcription and translation, impacting PAI-1 levels at the site of injury [42]. Third, PAI-1 activity cannot detect PAI-1 complexed to LRP1 found on smooth muscle cells and endothelial cells as they are no longer in circulation. Finally, since ISR occurs months following intervention, it remains unclear if baseline levels alone would be as predictive as repeated measurements. Repeat measurements of PAI-1 levels in these patients which would provide a comprehensive assessment of temporal PAI-1 levels from baseline to follow-up angiography or re-intervention. Nonetheless, despite these limitations we were able to link basal PAI-1 levels and restenosis. Future studies looking at modern revascularization techniques and temporal patterns of PAI-1 are warranted.

The value of PAI-1 as a biomarker has been questioned. First, PAI-1 expression is influenced by multiple pro-inflammatory conditions and is associated with various cardiovascular risk factors [6, 43]. For example, metabolic syndrome, obesity [44] and hyperinsulinemia [9]/insulin resistance [10] have all been linked with increased PAI-1 levels. In adjusted analysis, the predictive ability of elevated PAI-1 levels has not been independent of other cardiovascular risk factors and its additive benefit in risk prediction models has been lacking [45]. For example, Yarmolinsky et al. [3] reported that patients with diabetes had a significantly higher level of plasma PAI-1, which was associated with MACE. However, diabetics are at increased risk of both index and recurrent events. Accordingly, further studies are needed in more homogenous populations to ascertain the performance of PAI-1 in each individual cohort.

Our study is not without limitations. Relevant data could not be obtained from certain studies and patient level data were not available. In addition, the broad inclusion criteria resulted in a heterogeneous study population with differing PAI-1 measurement techniques. Variations in assays and standardizations as well as natural variations in PAI-1 levels may have influenced our results. For instance, considerable PAI-1 diurnal changes have been observed in previous studies [46, 47]. Most selected studies were of modest sample size and of low or moderate quality with only seven studies deemed to be of high quality [48]. The small number of studies may have limited the detection of small study effects or publication bias in funnel plots [49]. Finally, the large variation in study dates (1991 to 2016) spans a broad range of pharmacologic and revascularization practices, particularly coronary stent development, the introduction of dual antiplatelet therapy, and broadening indications for oral anticoagulation therapy. Accordingly, these findings may not be applicable in patients with specific risk profiles or those on contemporary medical therapy. Finally, while our study is provocative in the association demonstrated interventional studies are needed to link PAI-1 levels mechanistically to MACE.

## Conclusion

PAI-1 plasma levels are promising markers for MACE; however, high quality studies in well-defined populations are still needed to robustly evaluate the performance of PAI-1 as a clinical biomarker. Whether PAI-1 is a bona fide therapeutic target remains to be established.

## Additional file

Additional file 1: Association between plasminogen activator inhibitor-1 and cardiovascular events: a systematic review and meta-analysis. (DOCX 4410 kb)

## Abbreviations

CAD: Coronary artery disease
MACE: Major adverse cardiovascular events
PAI-1: Plasminogen activator inhibitor-1
PCI: Percutaneous coronary intervention
t-PA: Tissue-type plasminogen activator
u-PA: Urokinase-type plasminogen activator
