# Supplementary material for: Association between plasminogen activator inhibitor-1 and cardiovascular events: a systematic review and meta-analysis
Source: Thromb J. 2018 Jun 5;16:12. doi: 10.1186/s12959-018-0166-4 (PMC5987541; doi:10.1186/s12959-018-0166-4)
Supplement: Supplementary file 1 — Association between plasminogen activator inhibitor-1 and cardiovascular events: a systematic review and meta-analysis. (DOCX 4410 kb) [file 12959_2018_166_MOESM1_ESM.docx]

**Supplemental Material**


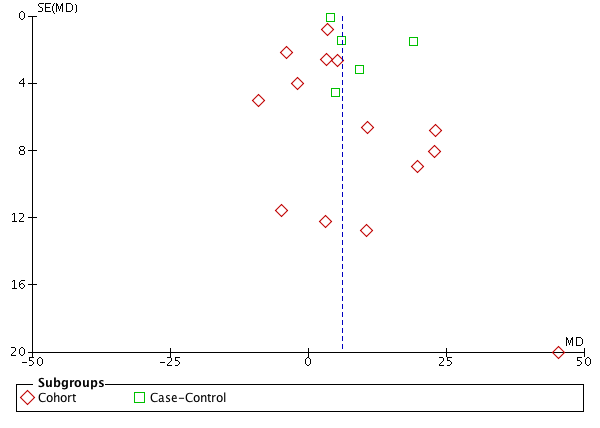


**Supplemental Figure 1.** Funnel plots for studies examining PAI-1 antigen levels (ng/mL) and MACE.


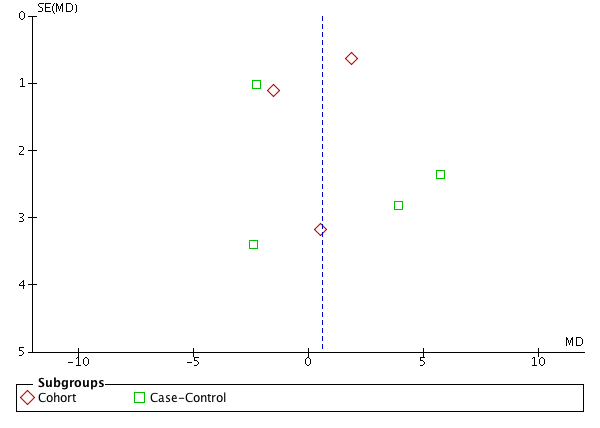


**Supplemental Figure 2.** Funnel plots for studies examining PAI-1 activity levels (IU/mL) and MACE.


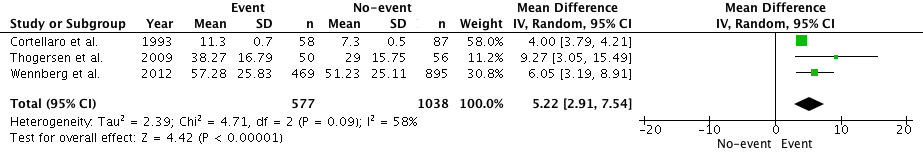
**Supplementary Figure 3.**  Comparison of mean PAI-1 antigen levels (ng/mL) and major adverse cardiac events in high-quality studies utilizing the Newcastle-Ottawa Scale.


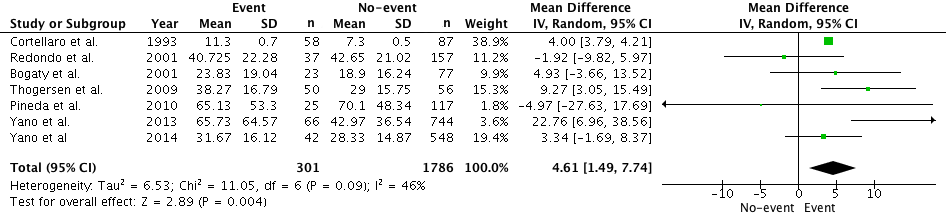


**Supplemental Figure 4.** Comparison of mean PAI-1 antigen levels (ng/mL) in studies which reported morning blood draws ranging from 7:00-10:00 am and major adverse cardiac events.

**
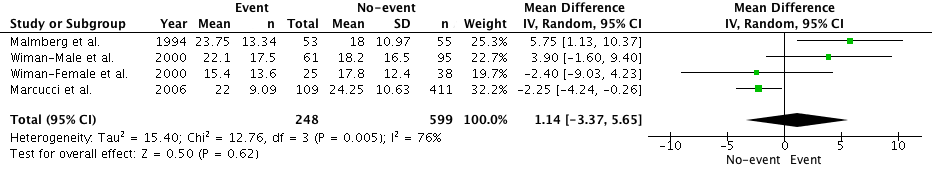
**

**Supplementary Figure 5.** Comparison of mean PAI-1 activity levels (IU/mL) and major adverse cardiac events in high-quality studies utilizing the Newcastle-Ottawa Scale.


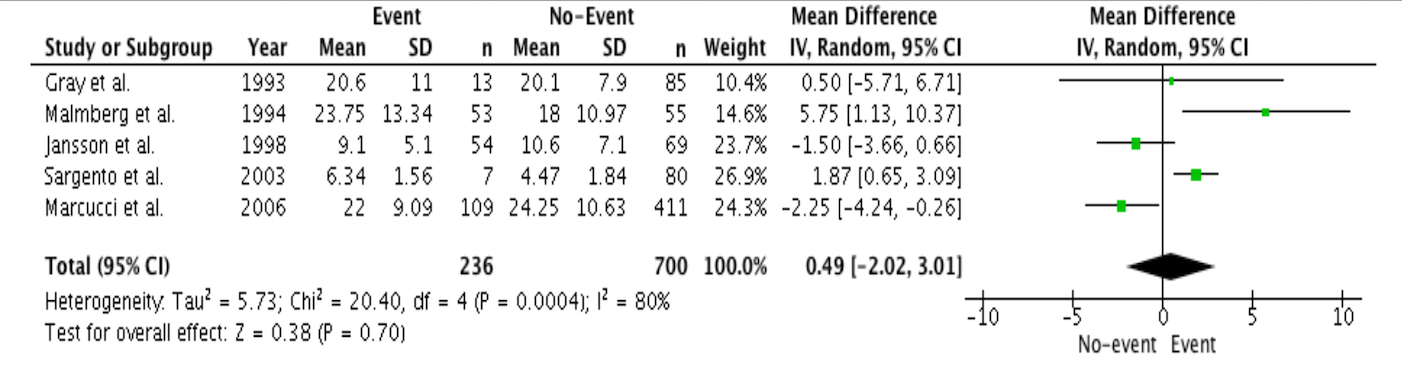


**Supplemental Figure 6.** Comparison of mean PAI-1 activity levels (IU/mL) in patients with secondary major adverse cardiac events and control patients.


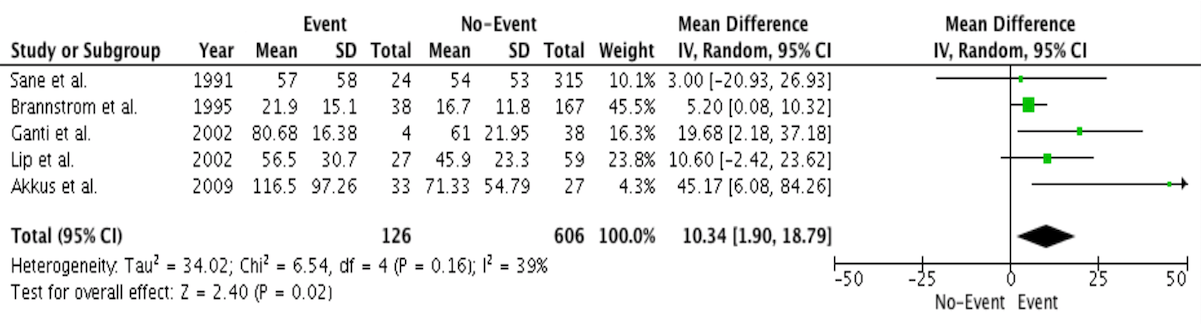


**Supplemental Figure 7.** Comparison of mean PAI-1 antigen levels (ng/mL) in event and control patients for mortality.

**Supplemental Table 1.** Newcastle-Ottawa Quality Assessment Scale for Observational Studies of PAI-1 antigen levels (ng/mL)

| **Reference** | **Year** | **Study Design** | **Selection** | **Comparability** | **Exposure/Outcome** | **Total** |
| --- | --- | --- | --- | --- | --- | --- |
| Sane et al.^[1]^ | 1991 | Cohort | 3 | 0 | 2 | 5 |
| Cortellaro et al.^[2]^ | 1993 | Case-Control | 2 | 2 | 3 | 7 |
| Brannstrom et al.^[3]^ | 1995 | Cohort | 3 | 0 | 3 | 6 |
| Juhan-Vague et al.^[4]^ | 1996 | Cohort | 3 | 0 | 3 | 6 |
| Nordt et al.^[5]^ | 1998 | Cohort | 3 | 0 | 3 | 6 |
| Alaigh et al.^[6]^ | 1998 | Cohort | 2 | 0 | 2 | 4 |
| Moss et al.^[7]^ | 1999 | Cohort | 2 | 0 | 3 | 5 |
| Redondo et al.^[8]^ | 2001 | Cohort | 3 | 0 | 2 | 5 |
| Fornitz et al.^[9]^ | 2001 | Cohort | 2 | 0 | 2 | 4 |
| Bogaty et al.^[10]^ | 2001 | Case-Control | 2 | 2 | 2 | 6 |
| Ganti et al.^[11]^ | 2002 | Cohort | 2 | 0 | 1 | 3 |
| Lip et al.^[12]^ | 2002 | Cohort | 3 | 0 | 3 | 6 |
| Inoue et al.^[13]^ | 2003 | Cohort | 3 | 0 | 2 | 5 |
| Christ et al.^[14]^ | 2005 | Cohort | 3 | 0 | 3 | 6 |
| El-Menyar et al.^[15]^ | 2006 | Cohort | 3 | 0 | 2 | 5 |
| Robinson et al.^[16]^ | 2007 | Cohort | 3 | 0 | 3 | 6 |
| Katsaros et al.^[17]^ | 2008 | Cohort | 3 | 0 | 2 | 5 |
| Thogersen et al.^[18]^ | 2009 | Case-Control | 4 | 2 | 2 | 8 |
| Akkus et al.^[19]^ | 2009 | Cohort | 3 | 0 | 2 | 5 |
| Arikan et al.^[20]^ | 2009 | Cohort | 3 | 0 | 3 | 6 |
| Pineda et al.^[21]^ | 2010 | Cohort | 3 | 0 | 3 | 6 |
| Wennberg et al.^[22]^ | 2012 | Case-Control | 4 | 2 | 3 | 9 |
| Yano et al. (2013)^[23]^ | 2013 | Cohort | 3 | 0 | 3 | 6 |
| Iacoviello et al.^[24]^ | 2013 | Cohort | 4 | 1 | 3 | 8 |
| Yano et al. (2014)^[25]^ | 2014 | Cohort | 3 | 0 | 3 | 6 |
| Knudsen et al.^[26]^ | 2014 | Case-Control | 2 | 2 | 1 | 5 |
| Golukhova et al.^[27]^ | 2015 | Cohort | 2 | 0 | 3 | 5 |

**Supplemental Table 2.** Newcastle-Ottawa Quality Assessment Scale for Observational Studies of PAI-1 activity levels (IU/mL)

| **Reference** | **Year** | **Study Design** | **Selection** | **Comparability** | **Exposure/Outcome** | **Total** |
| --- | --- | --- | --- | --- | --- | --- |
| Shah et al.^[28]^ | 1992 | Cohort | 3 | 0 | 2 | 5 |
| Gray et al.^[29]^ | 1993 | Cohort | 2 | 0 | 2 | 4 |
| Malmberg et al.^[30]^ | 1994 | Case-Control | 3 | 2 | 3 | 8 |
| Brack et al.^[31]^ | 1994 | Cohort | 3 | 0 | 1 | 4 |
| Jansson et al.^[32]^ | 1998 | Cohort | 1 | 0 | 3 | 4 |
| Sinkovic et al.^[33]^ | 2000 | Cohort | 2 | 0 | 2 | 4 |
| Wiman et al.^[34]^ | 2000 | Case-Control | 3 | 2 | 3 | 8 |
| Prisco et al.^[35]^ | 2001 | Cohort | 2 | 0 | 3 | 5 |
| Sargento et al.^[36]^ | 2003 | Cohort | 2 | 0 | 3 | 5 |
| Marcucci et al.^[37]^ | 2006 | Case-Control | 2 | 2 | 3 | 7 |
| Schoebel et al.^[38]^ | 2008 | Cohort | 2 | 0 | 3 | 5 |

**Supplemental Table 3.** Meta-regression results or the effect of time of blood draw and acute phase reaction on PAI-1 antigen and activity levels

|  |  | **PAI-1 antigen levels (ng/mL)** | | | | | | | | **PAI-1 activity levels (IU/mL)** | | | | | | |
| --- | --- | --- | --- | --- | --- | --- | --- | --- | --- | --- | --- | --- | --- | --- | --- | --- |
| **Model** | | | **N** | **Coeff.** | **95% CI** | **Tau2** | **Adj-R^2^(%)** | **I^2^ (%)** | **p** | **N** | **Coeff.** | **95% CI** | **Tau2** | **Adj-R^2^(%)** | **I^2^ (%)** | **p** |
| **All covariates** | | | 24 |  |  | 65.98 | -11.22 | 93.22 | 0.72 | 14 |  |  | 4.502 | -35.39 | 55.86 | 0.9 |
| Time of blood draw | | | 24 | -3.07 | (-12.60, 6.47) |  |  |  | 0.51 | 14 | 0.54 | (-3.15, 4.22) |  |  |  |  |
| Acute phase | | | 24 | 1.66 | (-9.35, 12.67) |  |  |  | 0.757 | 14 | 0.54 | (-3.06, 4.15) |  |  |  |  |

**Supplemental Methods** – Search Strategy

1. exp Myocardial Ischemia/ 388155
2. (myocard* adj5 (ischaemia or ischemia or infact*)).tw. 33300
3. ami.tw. 15268
4. (heart adj5 infarct*).tw. 12734
5. (isch?emi* adj5 heart).tw. 42573
6. exp Coronary Artery Bypass/ 48558
7. cabg.tw. 14694
8. CAG.tw. 8346
9. angina?.tw. 49387
10. (coronary adj5 (disease* or bypass or thrombo* or angioplast*)).tw. 176701
11. chd.tw. 19903
12. acute coronary syndrom*.tw. 22840
13. acs.tw. 14826
14. exp Percutaneous Coronary Intervention/ 42646
15. (percutaneous coronary adj2 (interven* or revascular*)).tw. 23160
16. pci.tw. 17415
17. ptca.tw. 6237
18. exp Angioplasty/ 57715
19. angioplast*.tw. 39128
20. ((coronary or arterial) adj4 dilat*).tw. 4969
21. endoluminal repair*.tw. 214\
22. exp Stents/ 62476
23. stent*.tw. 77393
24. exp Atherectomy/ 2101
25. atherectom*.tw. 2440
26. 1 or 2 or 3 or 4 or 5 or 6 or 7 or 8 or 9 or 10 or 11 or 12 or 13 or 14 or 15 or 16 or 17 or 18 or 19 or 20 or 21 or 22 or 23 or 24 or 25 603293
27. Plasminogen Activator Inhibitor 1/ 8107
28. PAI-1.tw. 9648
29. Plasminogen activator inhibitor-1.tw. 7085
30. 27 or 28 or 29 13351
31. 26 and 30 1405
32. Animals/ 5978308
33. Humans/ 16323582
34. 32 not 33 4282624
35. 31 not 34 1315

**Citations**

1. Sane DC, Stump DC, Topol EJ, Sigmon KN, Kereiakes DJ, George BS, Mantell SJ, Macy E, Collen D, Califf RM: **Correlation between baseline plasminogen activator inhibitor levels and clinical outcome during therapy with tissue plasminogen activator for acute myocardial infarction.** *Thrombosis and haemostasis* 1991, **65:**275-279.

2. Cortellaro M, Cofrancesco E, Boschetti C, Mussoni L, Donati MB, Cardillo M, Catalano M, Gabrielli L, Lombardi B, Specchia G, et al: **Increased Fibrin Turnover and High Pai-1 Activity as Predictors of Ischemic Events in Atherosclerotic Patients - a Case-Control Study.** *Arterioscler Thromb* 1993, **13:**1412-1417.

3. Brannstrom M, Jansson JH, Boman K, Nilsson TK: **Endothelial Hemostatic Factors May Be Associated with Mortality in Patients on Long-Term Anticoagulant Treatment.** *Thromb Haemost* 1995, **74:**612-615.

4. JuhanVague I, Pyke SDM, Alessi MC, Jespersen J, Haverkate F, Thompson SG: **Fibrinolytic factors and the risk of myocardial infarction or sudden death in patients with angina pectoris.** *Circulation* 1996, **94:**2057-2063.

5. Nordt TK, Moser M, Kohler B, Ruef J, Peter K, Kubler W, Bode C: **Augmented platelet aggregation as predictor of reocclusion after thrombolysis in acute myocardial infarction.** *Thromb Haemost* 1998, **80:**881-886.

6. Alaigh P, Hoffman CJ, Korlipara G, Neuroth A, Dervan JP, Lawson WE, Hultin MB: **Lipoprotein(a) level does not predict restenosis after percutaneous transluminal coronary angioplasty.** *Arterioscler Thromb Vasc Biol* 1998, **18:**1281-1286.

7. Moss AJ, Goldstein RE, Marder VJ, Sparks CE, Oakes D, Greenberg H, Weiss HJ, Zareba W, Brown MW, Liang CS, et al: **Thrombogenic factors and recurrent coronary events.** *Circulation* 1999, **99:**2517-2522.

8. Redondo M, Carroll VA, Mauron T, Demarmels Biasiutti F, Binder BR, Lammle B, Wuillemin WA: **Hemostatic and fibrinolytic parameters in survivors of myocardial infarction: A low plasma level of plasmin-alpha2-antiplasmin complex is an independent predictor of coronary re-events.** *Blood Coagulation and Fibrinolysis* 2001, **12:**17-24.

9. Fornitz GG, Nielsen P, Amtorp O, Kassis E, Abildgard U, Sloth C, Winther K, Orskov H, Dalsgard J, Husted S: **Impaired fibrinolysis determines the outcome of percutaneus transluminal coronary angioplasty (PTCA).** *Eur J Clin Invest* 2001, **31:**586-592.

10. Bogaty P, Poirier P, Simard S, Boyer L, Solymoss S, Dagenais GR: **Biological profiles in subjects with recurrent acute coronary events compared with subjects with long-standing stable angina.** *Circulation* 2001, **103:**3062-3068.

11. Ganti AK, Potti A, Yegnanarayan R: **Plasma tissue plasminogen activator and plasminogen activator inhibitor-1 levels in acute myocardial infarction.** *Pathophysiol Haemost Thromb* 2002, **32:**80-84.

12. Lip GYH, Blann AD, Farooqi IS, Zarifis J, Sagar G, Beevers DG: **Sequential alterations in haemorheology, endothelial dysfunction, platelet activation and thrombogenesis in relation to prognosis following acute stroke: The West Birmingham Stroke Project.** *Blood Coagul Fibrinolysis* 2002, **13:**339-347.

13. Inoue T, Yaguchi I, Mizoguchi K, Uchida T, Takayanagi K, Hayashi T, Morooka S, Eguchi Y: **Accelerated plasminogen activator inhibitor may prevent late restenosis after coronary stenting in acute myocardial infarction.** *Clin Cardiol* 2003, **26:**153-157.

14. Christ G, Nikfardjam M, Huber-Beckmann R, Gottsauner-Wolf M, Glogar D, Binder BR, Wojta J, Huber K: **Predictive value of plasma plasminogen activator inhibitor-1 for coronary restenosis: dependence on stent implantation and antithrombotic medication.** *J Thromb Haemost* 2005, **3:**233-239.

15. El-Menyar AA, Altamimi OM, Gomaa MM, Dabdoob W, Abbas AA, Abdel Rahman MO, Bener A, Albinali HA: **Clinical and biochemical predictors affect the choice and the short-term outcomes of different thrombolytic agents in acute myocardial infarction.** *Coronary artery disease* 2006, **17:**431-437.

16. Robinson SD, Ludlam CA, Boon NA, Newby DE: **Endothelial fibrinolytic capacity predicts future adverse cardiovascular events in patients with coronary heart disease.** *Arterioscler Thromb Vasc Biol* 2007, **27:**1651-1656.

17. Katsaros KM, Speidl WS, Kastl SP, Zorn G, Huber K, Maurer G, Glogar D, Wojta J, Christ G: **Plasminogen activator inhibitor-1 predicts coronary in-stent restenosis of drug-eluting stents.** *J Thromb Haemost* 2008, **6:**508-513.

18. Thogersen AM, Nilsson TK, Weinehall L, Boman K, Eliasson M, Hallmans G, Jansson J-H: **Changes in plasma C-reactive protein and hemostatic factors prior to and after a first myocardial infarction with a median follow-up time of 8 years.** *Blood coagulation & fibrinolysis : an international journal in haemostasis and thrombosis* 2009, **20:**340-346.

19. Akkus MN, Polat G, Yurtdas M, Akcay B, Ercetin N, Cicek D, Doven O, Sucu N: **Admission Levels of C-Reactive Protein and Plasminogen Activator Inhibitor-1 in Patients With Acute Myocardial Infarction With and Without Cardiogenic Shock or Heart Failure on Admission.** *Int Heart J* 2009, **50:**33-45.

20. Arikan H, Koc M, Tuglular S, Ozener C, Akoglu E: **Elevated Plasma Levels of PAI-1 Predict Cardiovascular Events and Cardiovascular Mortality in Prevalent Peritoneal Dialysis Patients.** *Ren Fail* 2009, **31:**438-445.

21. Pineda J, Marin F, Marco P, Roldan V, Valencia J, Ruiz-Nodar JM, Sogorb F, Lip GYH: **Premature coronary artery disease in young (age < 45) subjects: Interactions of lipid profile, thrombophilic and haemostatic markers.** *Int J Cardiol* 2009, **136:**222-225.

22. Wennberg P, Wensley F, Di Angelantonio E, Johansson L, Boman K, Rumley A, Lowe G, Hallmans G, Danesh J, Jansson JH: **Haemostatic and inflammatory markers are independently associated with myocardial infarction in men and women.** *Thromb Res* 2012, **129:**68-73.

23. Yano Y, Hoshide S, Shimada K, Kario K: **The Impact of Cigarette Smoking on 24-Hour Blood Pressure, Inflammatory and Hemostatic Activity, and Cardiovascular Risk in Japanese Hypertensive Patients.** *J Clin Hypertens* 2013, **15:**234-240.

24. Iacoviello L, Agnoli C, De Curtis A, di Castelnuovo A, Giurdanella MC, Krogh V, Mattiello A, Matullo G, Sacerdote C, Tumino R, et al: **Type 1 plasminogen activator inhibitor as a common risk factor for cancer and ischaemic vascular disease: the EPICOR study.** *BMJ Open* 2013, **3**.

25. Yano Y, Nakazato M, Toshinai K, Inokuchi T, Matsuda S, Hidaka T, Hayakawa M, Kangawa K, Shimada K, Kario K: **Circulating Des-acyl Ghrelin Improves Cardiovascular Risk Prediction in Older Hypertensive Patients.** *Am J Hypertens* 2014, **27:**727-733.

26. Knudsen A, Katzenstein TL, Benfield T, Jorgensen NR, Kronborg G, Gerstoft J, Obel N, Kjaer A, Lebech A-M: **Plasma plasminogen activator inhibitor-1 predicts myocardial infarction in HIV-1-infected individuals.** *AIDS (London, England)* 2014, **28:**1171-1179.

27. Golukhova EZ, Grigorian MV, Ryabinina MN, Bulaeva NI, Fortmann S, Serebruany VL: **Independent Predictors of Major Adverse Events following Coronary Stenting over 28 Months of Follow-Up.** *Cardiology* 2015, **132:**176-181.

28. Shah PK, Amin J: **Low high density lipoprotein level is associated with increased restenosis rate after coronary angioplasty.** *Circulation* 1992, **85:**1279-1285.

29. Gray RP, Yudkin JS, Patterson DL: **Enzymatic evidence of impaired reperfusion in diabetic patients after thrombolytic therapy for acute myocardial infarction: a role for plasminogen activator inhibitor?** *British heart journal* 1993, **70:**530-536.

30. Malmberg K, Bavenholm P, Hamsten A: **Clinical and biochemical factors associated with prognosis after myocardial infarction at a young age.** *Journal of the American College of Cardiology* 1994, **24:**592-599.

31. Brack MJ, More RS, Pringle S, Gershlick AH: **Absence of a Prothrombotic State in Restenotic Patients.** *Coronary Artery Dis* 1994, **5:**501-506.

32. Jansson JH, Nilsson TK, Johnson O: **von Willebrand factor, tissue plasminogen activator, and dehydroepiandrosterone sulphate predict cardiovascular death in a 10 year follow up of survivors of acute myocardial infarction.** *Heart (British Cardiac Society)* 1998, **80:**334-337.

33. Sinkovic A: **Prognostic role of plasminogen-activator-inhibitor-1 levels in treatment with streptokinase of patients with acute myocardial infarction.** *Clin Cardiol* 2000, **23:**486-489.

34. Wiman B, Andersson T, Hallqvist J, Reuterwall C, Ahlbom A, deFaire U: **Plasma levels of tissue plasminogen activator/plasminogen activator inhibitor-1 complex and von willebrand factor are significant risk markers for recurrent myocardial infarction in the Stockholm Heart Epidemiology Program (SHEEP) study.** *Arterioscler Thromb Vasc Biol* 2000, **20:**2019-2023.

35. Prisco D, Antonucci E, Fedi S, Margheri M, Giglioli C, Comeglio M, Lombardi A, Chioccioli M, Abbate R, Gensini GF: **D-Dimer increase after percutaneous transluminal angioplasty and clinical recurrence after primary revascularization in acute myocardial infarction? A pilot study.** *Clin Exper Med* 2001, **1:**219-224.

36. Sargento L, Saldanha C, Monteiro J, Perdigao C, Martins e Silva J: **Evidence of prolonged disturbances in the haemostatic, hemorheologic and inflammatory profiles in transmural myocardial infarction survivors.** *Thrombosis and haemostasis* 2003, **89:**892-903.

37. Marcucci R, Brogi D, Sofi F, Giglioli C, Valente S, Liotta AA, Lenti M, Gori AM, Prisco D, Abbate R, Gensini GF: **PAI-1 and homocysteine, but not lipoprotein (a) and thrombophilic polymorphisms, are independently associated with the occurrence of major adverse cardiac events after successful coronary stenting.** *Heart* 2006, **92:**377-381.

38. Schoebel FC, Peters AJ, Kreis I, Gradaus F, Heins M, Jax TW: **Relevance of hemostasis on restenosis in clinically stable patients undergoing elective PTCA.** *Thromb Res* 2008, **122:**229-236.
